# Effect of chemotherapy on hippocampal volume and shape in older long-term breast cancer survivors

**DOI:** 10.3389/fnagi.2024.1347721

**Published:** 2024-03-08

**Authors:** Ebenezer Daniel, Frank Deng, Sunita K. Patel, Mina S. Sedrak, Jonathan Young, Heeyoung Kim, Marianne Razavi, Can-Lan Sun, James C. Root, Tim A. Ahles, William Dale, Bihong T. Chen

**Affiliations:** ^1^Department of Diagnostic Radiology, City of Hope National Medical Center, Duarte, CA, United States; ^2^Department of Population Science, City of Hope National Medical Center, Duarte, CA, United States; ^3^Department of Medicine, Division of Hematology-Oncology, University of California Los Angeles David Geffen School of Medicine, Los Angeles, CA, United States; ^4^Center for Cancer and Aging, City of Hope National Medical Center, Duarte, CA, United States; ^5^Department of Supportive Care Medicine, City of Hope National Medical Center, Duarte, CA, United States; ^6^Neurocognitive Research Laboratory, Memorial Sloan Kettering Cancer Center, New York, NY, United States

**Keywords:** hippocampal volume, hippocampal shape, breast cancer, cancer-related cognitive impairment, chemotherapy

## Abstract

**Purpose:**

The objective of this study was to assess changes in hippocampal volume and shape in older long-term breast cancer survivors who were exposed to chemotherapy 5–15 years prior.

**Methods:**

This study recruited female long-term breast cancer survivors aged 65 years or older with a history of chemotherapy (C+), age-matched breast cancer survivors who did not receive chemotherapy (C−), and healthy controls (HC). The participants were recruited 5–15 years after chemotherapy at time point 1 (TP1) and were followed up for 2 years at time point 2 (TP2). Assessments included hippocampal volume and shape from brain MRI scans and neuropsychological (NP) tests.

**Results:**

At TP1, each of the three groups was comprised of 20 participants. The C+ group exhibited a hippocampal volume loss estimated in proportion with total intracranial volume (ICV) in both the left and right hemispheres from TP1 to TP2. Regarding the hippocampal shape at TP1, the C+ group displayed inward changes compared to the control groups. Within the C+ group, changes in right hippocampal volume adjusted with ICV were positively correlated with crystalized composite scores (*R* = 0.450, *p* = 0.044). Additionally, in C+ groups, chronological age was negatively correlated with right hippocampal volume adjusted with ICV (*R* = −0.585, *p* = 0.007).

**Conclusion:**

The observed hippocampal volume reduction and inward shape deformation within the C+ group may serve as neural basis for cognitive changes in older long-term breast cancer survivors with history of chemotherapy treatment.

## Highlights

• This study assessed hippocampal volume and shape alterations in older long-term breast cancer survivors who were exposed to chemotherapy 5–15 years prior.
• The chemotherapy-treated group showed a volume loss in bilateral hippocampi during the 2-year study duration.
• The chemotherapy-treated group displayed inward deformations/shrinkage as compared to the healthy control group in bilateral hippocampi upon enrollment at time point 1.
• Changes in right hippocampal volume were positively correlated with crystalized composite scores in the chemotherapy-treated group.

## 1 Introduction

Cancer-related cognitive impairment (CRCI) referring to cognitive difficulties experienced by cancer patients and survivors, is an increasing concern, specifically among older adults (Van Dyk and Ganz, 2021). Although there has been significant progress in cancer treatment, including targeted therapies and immunotherapy with improved prognosis in cancer survivors, CRCI remains an issue in survivorship affecting quality of life especially in older survivors (Országhová et al., 2021). Notably, older breast cancer survivors might experience persistent CRCI, particularly in learning and memory, years after treatment (Ahles et al., 2022). Cognitive dysfunction is of concern because it may compromise ability to perform functional tasks in daily living and adversely impacts quality of life. Several factors may contribute to CRCI, including the cancer itself, inflammation, chemotherapy, radiotherapy, hormonal therapy, and advancing age (Vega et al., 2017). Prior studies have implicated chemotherapy as a key reason for CRCI in women treated for breast cancer, suggesting treatment is associated with functional and structural changes in the brain. However, there is limited research focusing on the long-term effects of chemotherapy on brain changes specifically in older breast cancer survivors.

Neuroimaging studies focusing on the hippocampus have played an important role in identifying biomarkers associated with CRCI in cancer survivors who received chemotherapy (Peukert et al., 2020). Previous work suggests that hippocampal structure may offer early indications of cognitive dysfunction and brain aging, particularly in older adults (O’Shea et al., 2016). Various studies have revealed hippocampal changes in breast cancer patients with volume loss (Kesler et al., 2013), shape alteration (Apple et al., 2017), and decreased functional connectivity (Apple et al., 2018). For instance, the study by Kesler et al. (2013) reported reduced left hippocampal volume being associated with impaired verbal memory function in a cohort of breast cancer patients ranging from 41 to 73 years of age at 1–12 years post-chemotherapy treatment. A study focusing on younger breast cancer survivors between the ages of 28–45 years, reported significant morphological differences in hippocampal structure, along with increased self-reported cognitive difficulties and worse episodic memory (Apple et al., 2017). Additionally, higher hippocampal-cortical connectivity was also noted in the breast cancer patients (Apple et al., 2018). These changes have been associated with CRCI, affecting cognitive domains such as executive function, working memory, episodic memory, and prospective memory (Peukert et al., 2020). Nevertheless, a prior study of long-term breast cancer survivors with a mean age of 64 years and on average of 21 years after chemotherapy reported no significant differences in hippocampal volume between the survivors with chemotherapy exposure and a population-based sample of women without a history of cancer (Koppelmans et al., 2012). Therefore, more studies are needed to characterize potential alterations of the hippocampus in long-term cancer survivors.

Regional specialization in the hippocampus has been noted, with the anterior part linked to the regulation of affect, stress, and emotions, while the posterior part linked to cognitive functions such as memory and spatial learning (Moser and Moser, 1998). Our own studies focusing on the acute effects of chemotherapy in older breast cancer patients have shown a decrease in gray matter density in the bilateral medial temporal lobes in patients receiving chemotherapy (Chen et al., 2018a), as well as a reduction in the total volume of the temporal lobes (Chen et al., 2018b). While existing literature has established the acute detrimental impact of chemotherapy on the hippocampus, its long-term effects on hippocampal volume and shape remain relatively unexplored, particularly in older cancer survivors.

Here, we conducted a prospective longitudinal study focusing on older long-term female breast cancer survivors with (C+) or without (C−) a history of chemotherapy with treatment completed 5–15 years prior, along with an age-matched healthy control (HC) group, followed over a 2-year period. We aimed to investigate the long-term changes in hippocampal volume and shape, and their potential associations with neuropsychological (NP) testing scores. We hypothesized that the chemotherapy-treated group would have lower hippocampal volume and altered shape with atrophy, which would correlate with NP testing scores. By focusing on older breast cancer survivors and long-term effects of chemotherapy, our study should provide the much-needed insights into neural correlates and CRCI in long-term older cancer survivors.

## 2 Methods

### 2.1 Participants

We recruited breast cancer survivors 5–15 year after treatment, aged 65 years or older, treated with (C+) or without (C−) chemotherapy, together with age and sex-matched healthy controls (HC) with no history of cancer at time point 1 (TP1). The age matching was done by frequency match on age (±5 years). All study participants were female, and the age variable was matched independently. Follow-up assessment was performed at a 2-year interval at time point 2 (TP2).

Participants with a history of psychiatric disease, other cancer diagnoses or treatments, neurodegenerative disorders, metastatic disease, or stroke were excluded from the study. The HC participants were recruited through patient referrals, local newspaper advertisements, and community health fairs. This neuroimaging study was conducted as a sub-study of the parent study titled “Cognition in older breast cancer survivors: treatment exposure, APOE, and smoking history” (NCT02122107), and written informed consent was obtained from all participants. The study was approved by the Institutional Review Board at City of Hope National Medical Center.

Clinical and demographic data were assessed through analysis of variance (ANOVA) for continuous variables, while Fisher’s exact tests were used for categorical variables. A threshold of *p*-value at 0.05 was considered significant for both continuous and categorical variables, with all tests conducted as two-sided assessments. The data analysis was performed with SAS 9.3 (SAS Institute, Cary, NC, USA).

### 2.2 MRI data acquisition

All brain MRI scans were acquired using the 3T VERIO Siemens scanner (Siemens, Erlangen, Germany). The scanning of T1-weighted 3-dimensional (3D) magnetization prepared rapid gradient echo (MPRAGE) image data was performed with the following parameters: TR (repetition time) = 1,900 ms; TE (echo time) = 2.94 ms; inversion time = 900 ms; FA (flip angle) = 9°; and voxel size of 0.45 mm × 0.45 mm × 1.5 mm.

### 2.3 NP testing

We conducted NP testing using the NIH Toolbox Cognition Battery (Gershon et al., 2013). This battery provided seven independent scores, evaluating various cognitive abilities, such as picture sequence memory, pattern comparison processing speed, picture vocabulary, oral reading recognition, list sorting working memory, flanker inhibitory control, and dimensional change card sorting. Additionally, we obtained composite scores for crystallized, fluid, and total composite function from the cognition battery. To address the issue of repeated measurements within subjects, we utilized a linear mixed model (LMM) for data analysis. The LMM allowed us to treat group (C+, C−, and HC) and time-point (TP1 and TP2) as categorical fixed effects (Laird and Ware, 1982; Daniel et al., 2023). The LMM was employed to investigate the following: (1) potential disparities in the NP scores among the three groups at TP1 or TP2; (2) significant longitudinal differences within each group; and (3) the presence of a group-by-time interaction effect. Data analysis was conducted using SAS 9.3 (SAS Institute, Cary, NC, USA).

### 2.4 Hippocampal volumetric analysis

We utilized the FIRST^1^ tool implemented on FSL v.6.0.6.5 (FMRIB’s Software Library) to automatically segment the left and right hippocampal regions individually from T1-weighted brain MRI images. All images were individually normalized to the default setting “first” in the Montreal Neurological Institute (MNI 152) space. Using the “fslstats” option within the “first” toolbox, hippocampal volumes (mm^3^) were calculated, with standard label numbers corresponding to the “first” toolbox (e.g., label numbers 17 for the left hippocampus and 53 for the right hippocampus). The extraction of hippocampal volumes involved selecting the single label number with a ±0.5 offset based on the standard label number 17 and 53, according to the procedure published previously (Patenaude et al., 2011). All the hippocampal volume date were adjusted with total intracranial volume (ICV) (Van Petten, 2004). The ICV of each participants was calculated with brain extraction tool (BET) using FSL software (Smith, 2002). The hippocampal volume was normalized after being divided by TIV in units of mm^3^ by multiplying with 10^6^ (Jack et al., 1998). To analyze the statistical differences in derived hippocampal volumes, we used the IBM Statistical Package for the Social Sciences (IBM SPSS, V 29, Chicago, IL, USA). The “independent sample *t*-test” was employed to test the differences between groups (C+ and C−, C+ and HC, and C− and HC) at TP1. To assess longitudinal changes within the groups over the 2-year period, we used the “paired *t*-test.” We investigated the group by time interaction among the three groups during the 2-year study duration using the mixed model analysis. There were no covariates included in the design matrix.

### 2.5 Hippocampal shape analysis

We employed vertex-based analysis with “first_utils” to calculate hippocampal shape indices. To assess the statistical differences, we used “randomize” with 5,000 random permutations implemented in the FSL software. The design matrix, t-contrast, and f-contrast were created using the “General Linear Model” (GLM) GUI in FSL software. Positive contrast results indicated outward hippocampal deformations (expansion), while negative contrast represented inward hippocampal deformation (shrinkage). We corrected the results for multiple comparisons using threshold-free cluster enhancement (TFCE) with a family-wise error (FWE) rate of *p* < 0.05 (Smith and Nichols, 2009; Patenaude et al., 2011; Wang et al., 2022).

### 2.6 Correlation analysis

A total of 14 correlation analyses were conducted in the study, comprising 8 analyses for TP1 and 6 analyses for longitudinal changes. At TP1, correlations were examined between the left and right hippocampal volumes expressed as proportion of the ICV and three neuropsychological composite scores (Fluid Cognition, Crystallized Cognition, and Total Cognition), along with the participants’ chronological age. Additionally, correlations were explored between the changes in hippocampal volume expressed as proportion of the ICV (both left and right hemisphere) and the changes in the three neuropsychological composite scores from TP1 to TP2. We used bivariate Pearson correlation and considered the *p*-values <0.05 being significant correlation. Multiple comparison correction was not applied. As all the participants were female, therefore we did not include sex as a covariate in our analysis. We did not adjust the age when testing the correlations, mostly due to the concern for such a small sample size in our study.

## 3 Results

### 3.1 Clinical and demographic data

We enrolled a cohort of 60 participants at TP1, with 20 participants in each of the three groups (C+, C−, and HC). At TP1, we performed cross-sectional analysis between the groups (C+ and HC, C+ and C−, and C− and HC). However, over the course of the study during the 2-year follow-up, we experienced significant attrition due to various issues from the participants including new memory problems, new cancer diagnoses, refusal to continue participation, loss to follow-up, and death. Hence, there were a total of 39 participants available for the TP2 analysis. For within-group longitudinal analysis from TP1 to TP2, there were 12 participants in the C+ and C− groups and 15 participants in the HC group. There were no differences in mean follow-up time between the C+ and C− groups based on the years from diagnosis to TP1 (*p* = 0.372). Table 1 presents demographic data for the participants, indicating no statistically significant differences in terms of age (*p* = 0.75), education (*p* = 0.80), or race (*p* = 0.37). More details can be found in our published studies focusing on cortical thickness (Daniel et al., 2023) and white matter microstructural changes (Daniel et al., 2024).

**TABLE 1:** Demographic and clinical information.

Parameters	TP1				Longitudinal data with both TP1 and TP2			
	C+ *N* = 20	C− *N* = 20	HC *N* = 20	*p*	C+ *N* = 12	C− *N* = 12	HC *N* = 15	*p*
**Age (years)**								
Mean (SD)	73.5 (5.06)	76.85 (4.63)	74.00 (6.09)	0.10	73.75 (5.41)	76.50 (4.28)	74.53 (6.73)	0.48
Median (range)	73.5 (66–84)	77.5 (69–86)	72.5 (66–88)		71.50 (68–84)	75.5 (71–86)	73.00 (66–88)	
**Race (*N*, %)**								
White or Caucasian	15 (75)	18 (90)	18 (90)	0.09	10 (83)	11 (92)	14 (93)	0.76
Black	1 (5)	2 (10)			1 (8)	1 (8)		
Asian or Native Hawaiian/Pacific Islander	4 (20)	–	1 (10)		1 (8)	–	1 (7)	
Unknown	–	–	1 (10)		–	–	–	
**Ethnicity (*N*, %)**								
Not Hispanic	18 (90)	20 (100)	17 (85)	0.35	10 (83)	12 (100)	13 (87)	0.53
Hispanic	2 (10)		3 (15)		2 (17)	–	2 (13)	
**Education (*N*, %)**								
High school or less	4 (20)	5 (25)	6 (30)	0.93	3 (25)	4 (33)	4 (27)	0.99
College or above	16 (80)	15 (75)	14 (70)		9 (75)	8 (67)	11 (73)	
**AJCC stage (*N*, %)**								
DCIS	1 (5)	9 (45)	–		1 (8)	6 (50)	–	
I	4 (20)	8 (40)	–		1 (8)	4 (33)	–	
II	14 (70)	3(15)	–		10 (83)	2 (17)	–	
III	1 (5)	–	–		–	–	–	
**Regimen**								
Non-Trastuzumab regimen (*N*, %)	16 (80)	–	–		9 (75)	–	–	
Trastuzumab regimen (*N*, %)	4 (20)	–	–		3 (25)	–	–	
Years from diagnosis to TP1 (mean, SD)	7.42 (2.34)	8.20 (2.86)						

TP1, time point 1; TP2, time point 2; C+, chemotherapy group; C−, no-chemotherapy group; HC, healthy control group; SD, standard deviation; AJCC, American Joint Committee on Cancer; DCIS, ductal carcinoma *in situ*; *N*, number of subjects. Statistical significance is set at *p*-value being <0.05. In the context of race, “Unknown” indicates that the patient declined to provide the data regarding race.

### 3.2 NP testing data

At TP1, the C+ group showed significantly lower scores in crystallized composite (*p* = 0.04) and oral reading recognition (*p* = 0.02) as compared to the C− group. Within the C+ group, longitudinal analysis revealed decreased total composite scores (*p* = 0.01), fluid composite scores (*p* = 0.03), and picture vocabulary scores (*p* = 0.04) over time. Moreover, the decreases in crystallized composite score (*p* = 0.057) and picture sequence memory scores (*p* = 0.05) were marginally significant in the C+ group. No significant differences in cognitive testing scores were observed within the control groups. Furthermore, there were no significant group-by-time differences in the cognitive testing scores. Detailed NP testing scores were reported in our prior study that focused on cortical thickness analysis (Daniel et al., 2023).

### 3.3 Hippocampal volumetric data

Regarding the hippocampal volume expressed as proportion of the ICV (mm^3^) and number of voxels in the left and the right hemispheres, there were no significant differences between the groups (C+ versus C−, C+ versus HC, and C− versus HC) at TP1 (all *p* > 0.05) (Tables 2, 3). The longitudinal within-group hippocampal volume from TP1 to TP2 adjusted as proportion of the ICV showed decreased left (*p* = 0.033) and right (*p* = 0.004) hippocampus volume within the C+ group. Whereas in the C− group, only the right hippocampus volume (*p* = 0.034) adjusted as proportion of the ICV was decreased from TP1 to TP2. No significant longitudinal changes were noted in the HC group from TP1 to TP2 at *p* > 0.05 (Table 4).

**TABLE 2:** Between-group comparison of hippocampal volume adjusted with total intracranial volume at time point 1.

Groups/parameters	*t*-Value	Degree of freedom (df)	CI (95%)		*p*-Value
			Lower	Upper	
**C+/HC**					
Volume in LH	1.82	24.65	−42.59	696.23	0.08
Number of voxels in LH	1.82	24.65	−140.71	2,300.09	0.08
Volume in RH	1.57	28.86	−82.01	624.05	0.13
Number of voxels in RH	1.57	28.86	−270.92	2,061.65	0.13
**C+/C−**					
Volume in LH	1.92	24.71	−24.45	714.86	0.07
Number of voxels in LH	1.92	24.71	−80.77	2,361.64	0.07
Volume in RH	1.16	24.42	−147.06	526.31	0.26
Number of voxels in RH	1.16	24.42	−485.85	1,738.73	0.26
**C−/HC**					
Volume in LH	0.20	38.00	−205.36	168.59	0.84
Number of voxels LH	0.20	38.00	−678.45	556.96	0.84
Volume in RH	0.82	34.56	−120.66	283.46	0.42
Number of voxels in RH	0.82	34.56	−398.60	936.45	0.42

CI, 95% of confidence interval; SD, standard deviation; LH, left hemisphere; RH, right hemisphere; C+, chemotherapy group; C−, no-chemotherapy control group; HC, healthy control group. *p*-Values significant at <0.05. The number of participants was 20 for each of the three groups at time point 1.

**TABLE 3:** Hippocampal volume data adjusted with total intracranial volume at time point 1.

Parameters	Mean (SD)		
	C+ group	C− group	HC group
Volume in LH (mm^3^)	2,181.44 (746.80)	2,526.64 (292.94)	2,508.26 (291.20)
Number of voxels in LH	7,206.69 (2,467.16)	8,347.12 (967.78)	8,286.38 (962.03)
Volume in RH (mm^3^)	2,440.25 (682.24)	2,629.87 (260.29)	2,711.27 (360.82)
Number of voxels in RH	8,061.71 (2,253.86)	8,688.15 (859.91)	8,957.07 (1,192.04)

SD, standard deviation; LH, left hemisphere; RH, right hemisphere; C+ group, chemotherapy group; C− group, no-chemotherapy control group; HC group, healthy control group. The number of participants was 20 for each of the three groups at time point 1.

**TABLE 4:** Longitudinal within-group differences in hippocampal volume adjusted with total intracranial volume.

Groups/parameters	Mean difference (SD) (TP1–TP2)	*t*-Value	CI (95%) of paired difference		*p*-Value
			Lower	Upper	
**Volume in LH**					
C+ group	68.12 (97.15)	2.4	6.40	129.84	0.033
C− group	77.86 (153.89)	1.7	−19.54	175.25	0.106
HC group	57.58 (175.74)	1.2	−48.62	163.78	0.260
**Number of voxels in LH**					
C+ group	225.04 (320.96)	2.4	21.11	428.96	0.033
C− group	257.21 (506.41)	1.7	−64.54	578.97	0.106
HC group	190.22 (580.58)	1.2	−160.62	541.06	0.260
**Volume in RH**					
C+ group	94.53 (91.43)	3.6	36.53	152.62	0.004
C− group	68.36 (98.04)	2.4	6.07	130.67	0.034
HC group	37.25 (141.69)	0.9	−48.37	122.87	0.362
**Number of voxels in RH**					
C+ group	312.29 (302.07)	3.6	120.37	504.22	0.004
C− group	225.84 (323.89)	2.4	20.05	431.63	0.034
HC group	123.06 (468.09)	0.9	−159.80	405.92	0.362

TP1, time point 1; TP2, time point 2; SD, standard deviation; CI, 95% of confidence interval; LH, left hemisphere; RH, right hemisphere; C+ group, chemotherapy group; C− group, no-chemotherapy control group; HC group, healthy control group. *p*-Values significant at <0.05.

### 3.4 Hippocampal shape analysis

**FIGURE 1:**
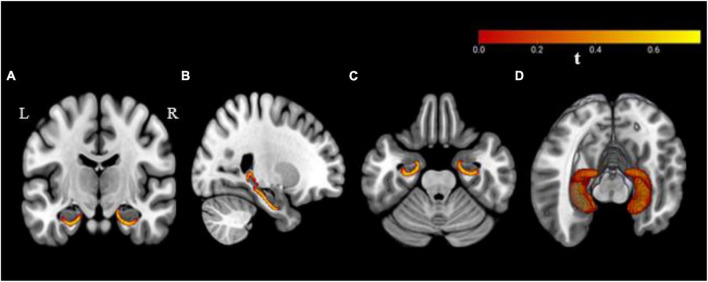
Bilateral hippocampal shape deformations in the chemotherapy group as compared to the healthy control group with significance at a threshold *p* ≤ 0.05 based on family-wise error (FWE) correction. Results are overlaid on a standard Montreal Neurological Institute (MNI 152) template, **(A)** coronal, **(B)** sagittal, **(C)** axial plane, and **(D)** three-dimensional view. R, right hemisphere; L, left hemisphere.

The vertex-wise analysis was performed to estimate the statistical shape deformations between the groups (C+ versus C−, C+ versus HC, and C− versus HC) at TP1. The C+ group showed inward shape deformation/shrinkage over HC group in bilateral hippocampi at TP1 (Figure 1). There was significant inward deformation/shrinkage in the left hippocampus in the C+ group as compared to the C− group. The C− group showed inward shape deformation in the right hippocampus over the HC group at TP1. The longitudinal analysis from within-group vertex-wise analysis showed no significant shape differences within the C+, C−, or the HC group during the 2-year interval. There were no significant hippocampal shape differences based on group-by-time interaction among the three groups. These results were obtained after multiple comparisons with FWE correction and statistical significance set at a threshold of 0.05.

### 3.5 Correlation data

**FIGURE 2:**
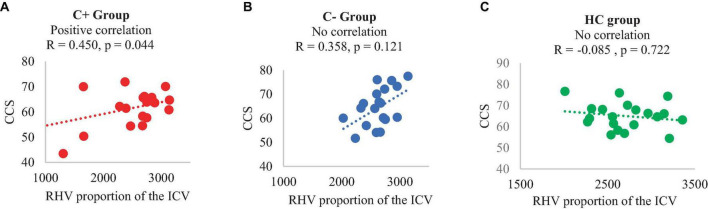
Correlation of right hippocampal volume (adjusted with total intracranial volume) and crystalized composite score at time point 1, **(A)** C+ group: chemotherapy group, **(B)** C− group: no-chemotherapy group, **(C)** HC group: healthy control group. CCS: crystallized composite score, RHV: right hippocampal volume in mm^3^, R: correlation coefficient with significance set at *p* < 0.05.

**FIGURE 3:**
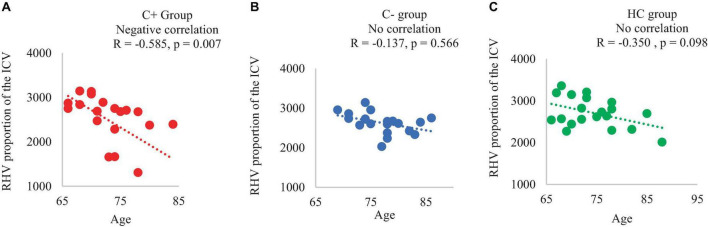
Correlation of right hippocampal volume adjusted with total intracranial volume (ICV) and chronological age of the participants at time point 1 **(A)** C+ group: chemotherapy group, **(B)** C− group: no-chemotherapy group, **(C)** HC group: healthy control group. RHV: right hippocampal volume in mm^3^, R: correlation coefficient with significance at *p* < 0.05.

In the C+ group, a significant positive correlation was found between the crystallized composite score and the right hippocampal volume adjusted as proportion of the ICV at TP1 (*p* = 0.044, *R* = 0.450). However, no significant correlations were observed in the C− or the HC group (Figure 2). Additionally, there was a negative correlation between the right hippocampal volume adjusted as proportion of the ICV and age in the C+ group at TP1 (*p* = 0.007, *R* = −0.585) (Figure 3). These results remained significant at a threshold of 0.05. No significant correlation was found between the longitudinal changes in hippocampal volume adjusted as proportion of the ICV and the changes in NP testing scores in any of the three group.

## 4 Discussion

In this study, we identified decreased hippocampal volume longitudinally and altered shape with shrinkage in the older long-term survivors of breast cancer who had chemotherapy treatment 5–15 years prior. We also found a significant positive correlation between hippocampal volume and cognitive testing scores in the chemotherapy-treated group. Additionally, we observed a negative correlation between right hippocampal volume and chronological age in the chemotherapy-treated group.

Hippocampus, as a part of the temporal lobe, plays a crucial role in cognitive functions such as learning and memory function (Anand and Dhikav, 2012). The effect of chemotherapy on the hippocampus has been studied in the context of CRCI. However, most of the prior studies were focused on the short-term impact of chemotherapy in breast cancer patients. For instance, one group reported right hippocampal volume loss in younger breast cancer patients (ages 18–45 years), assessing the short-term impact of CRCI after 18 months of chemotherapy treatment (Apple et al., 2017). Another study identified left hippocampal volume loss in breast cancer survivors aged 41–73 years, 1–12 years following chemotherapy (Kesler et al., 2013). A study showing both left and right hippocampal volume loss was performed on individuals aged 18–55 years, assessing the impact of radiation therapy on cognition 18–36 months after treatment in breast cancer patients (Bergouignan et al., 2011). Our own prior studies have shown reduced temporal lobe volume within months after chemotherapy in older breast cancer (Chen et al., 2018a,b). The literature on hippocampal volume in older long-term survivors has been limited. A study of long-term breast cancer survivors with a mean age of 64.0 (SD = 6.5) years and an average of 21 years post-treatment reported no significant difference in hippocampal volume between the survivors and the population controls (Koppelmans et al., 2012). The present study focusing on older breast cancer survivors (68–84 years of age) also showed no significant differences in hippocampal volume between the C+ group and the control groups at TP1. Moreover, our longitudinal study design enabled us to assess hippocampal volume changes over a 2-year interval, contributing new data on hippocampal alterations in older cancer survivors. The observed left and right hippocampal volume loss in our C+ group implicated it as a potential neural correlate of long-term effect of chemotherapy in older breast cancer survivors.

Both the left and the right hippocampi play important role in memory processes, but their specific functions differ (Miller et al., 2018). The left hippocampus is important in episodic verbal memory, while the right hippocampus is involved in the processing of spatial memory (Ezzati et al., 2016). The functional differences between the left and the right hemisphere may have structural correlates in hippocampal morphology in cancer survivors. For instance, a prior study showed a significant volume reduction in the left hippocampus only, which was associated with impaired verbal memory function in breast cancer survivors post-chemotherapy treatment (Kesler et al., 2013). Another study of breast cancer survivors showed only the right hippocampal volume loss in the chemotherapy group as compared to control groups, which was associated with increased self-reported cognitive impairment and worse episodic memory (Apple et al., 2017). In the current study, we also identified bilateral hippocampal shape alterations in the C+ group at 5–15 years after chemotherapy treatment. We speculate that these morphological changes may have initiated during the early stages of post-chemotherapy and persist into the long term. A previous study (Apple et al., 2017) showing hippocampal deformation within 18 months of chemotherapy supported our speculation.

The effect of aging on CRCI might be more pronounced in older survivors (Loh et al., 2016). A prior study showed that hippocampal atrophy was associated with memory impairment including episodic memory, semantic memory, and working memory during late adulthood (Dawe et al., 2020). Alterations in other brain areas have also been shown to be correlated with cognitive impairment and accelerated aging in patients exposed to chemotherapy. For instance, our own study on the the same cohort of older patients showed cortical thinning in the temporal lobe, specifically in the fusiform gyrus and inferior temporal regions in the chemotherapy-treated breast cancer survivors (Daniel et al., 2023). Other researchers also noted a significant decrease in cortical thickness and an incrase in predicted brain age in the breast cancer patients after chemotherapy as compared to the controls, which was associated with diminished memory performance (Henneghan et al., 2020). In our earlier study on a different cohort of older breast cancer patients, we found a reduction of gray matter density in the left anterior cingulate gyrus, bilateral insula, bilateral middle temporal gyri, and left parahippocampal gyrus shortly after chemotherapy (Chen et al., 2018a,b). In addition, hippocampal volume loss and its correlation with episodic and working memory function were noted in a group of healthy older adults 65–88 years of age (Hardcastle et al., 2020). Furthermore, hippocampal volume loss has been well documented in patients with Alzheimer’s dementia (AD), mild cognitive impairment and individuals with normal aging (Jack et al., 2000; Dawe et al., 2020; Hardcastle et al., 2020). In neurodegenerative studies, hippocampal volume reduction is identified as a biomarker for episodic memory deficits and for progression of AD (Jack et al., 2000; Driscoll et al., 2003). In our cohort, we observed lowered memory scores including the picture sequence memory and the fluid composite score. However, we did not find any significant correlation between hippocampal volume and episodic memory deficits in our C+ group. We speculate that it might be due to the small sample size of our study. A larger cohort may be needed to test the correlation of hippocampal volume loss and episodic memory impairment in long-term older breast cancer survivors.

We observed the inward deformation, i.e., shrinkage, of the right hippocampus in the cancer survivors after chemotherapy treatment. The right hippocampal deformation in our breast cancer survivors aligned with a previous study of short-term effects of chemotherapy on younger cancer survivors (Apple et al., 2017). This implied that the hippocampal shape deformation noted in the short-term (6–18 months) after chemotherapy treatment could potentially persist into long-term (5–15 years) after chemotherapy in older breast cancer survivors. A study of longitudinal gray matter changes within 1 year after chemotherapy treatment reported decreased right hippocampal gray matter density in breast cancer patients aged 18–65 years who received chemotherapy (Lepage et al., 2014). Moreover, earlier research of brain gray matter revealed lack of recovery in the temporal lobe including the hippocampal areas while sufficient recovery in the frontal lobe regions at the 1-year follow-up (McDonald et al., 2010). Therefore, we speculate that the hippocampal morphological deformation observed in the present study may have occurred during the early post-chemotherapy period and may have persisted years after treatment. Nevertheless, further studies including prior to, during, and after chemotherapy are needed to investigate the trajectory of hippocampal changes over time. We also found the right hippocampal volume reduction over 2 years in the survivors without chemotherapy. We speculate that these volume changes could be due to hormonal treatment since most patients received endocrine therapy in the no-chemotherapy group and endocrine therapy has been reported as being associated with CRCI (Van Dyk et al., 2019). Nevertheless, we could not tease out the effect of hormonal therapy on hippocampal volume in this study due to the small sample size. Future large studies with sufficient statistical power are needed to take into consideration of various confounding variables such as treatment strategies.

We identified a significant positive correlation between the right hippocampal volume and the crystalized composite score in the C+ group, implicating the hippocampal changes being relevant to cognitive function even 5–15 years after chemotherapy treatment. However, we found no correlation between the changes in hippocampal volume and the changes in the NP scores, which was not unexpected. Crystallized composite score assesses learned knowledge and cognitive skills, which has been a dependable cognitive ability that tends to be maintained with age (Akshoomoff et al., 2013; Bajpai et al., 2022). Our own research focusing on cortical thickness and white matter microstructure also showed the resilience of crystallized composite score to change and the lack of correlation between this NP score and brain structural changes in chemotherapy-treated older cancer survivors (Daniel et al., 2023, 2024). Additionally, we found a more significant negative association between the right hippocampal volume and the chronological age in the chemotherapy group over the control groups. This result implied that the hippocampal loss might be more pronounced with aging in the chemotherapy group.

There were several limitations to this study. First, it was a neuroimaging pilot study with a small sample size, and there was significant attrition over the 2-year follow-up period. In addition, this study was limited by the lack of diversity of our study cohort with most participants being Caucasian, which limited the generalizability of data to other racial groups. We will work on enrolling a more diversified cohort encompassing all racial groups in our future studies. Second, we found shape differences but no volume differences at TP1, which we speculate might be due to shape changes being more sensitive to volume changes. Similar findings were noted on a prior study of patients with major depressive disorder as compared to healthy controls (Posener et al., 2003). In that study, the researchers using high-dimensional brain mapping method identified significant differences in a series of 10 variables representing hippocampal shape between the depressed patients and the control subjects. However, they found no between group differences in hippocampal volume (Posener et al., 2003). In our study, we found within-group changes from TP1 to TP2 in volume but not in shape. We speculate that it might be partly due to the “paired *t*-test” used for longitudinal within-group analysis being more sensitive than “independent sample *t*-test” used for TP1 analysis for assessing volume changes in a small sample size. Third, our study only evaluated longitudinal changes over a 2-year interval during survivorship. The trajectory of hippocampal changes prior to chemotherapy, shortly after chemotherapy and long-term survivorship could not be assessed from our study design. Finally, this pilot study did not examine the hippocampal subfields due to the small sample size. Detailed assessment of the subfields is important given the regional specialization of the hippocampus with the posterior part being more involved in cognitive function. This study was also limited as the correlation analysis was not adjusted for age. Future studies with a larger sample size are needed to account for these variables. Despite these limitations, our study has strengths. It was the first longitudinal study of hippocampal volume and shape in older long-term survivors with history of chemotherapy, which should advance our understanding of CRCI in older cancer survivors. This pilot observational study should provide preliminary data to power a large-scale prospective longitudinal study, aiming to identify neuroimaging biomarkers for CRCI in older cancer survivors. This work should also shed light on the need for more research on older adults to improve their cognitive function and quality of life.

## 5 Conclusion

In summary, we found the hippocampal shape alteration in the chemotherapy group over the control groups at TP1. In addition, we also identified reduced bilateral hippocampal volume longitudinally during the 2 years of follow-up in older long-term breast cancer survivors who received chemotherapy many years ago. Furthermore, we identified a significant correlation among hippocampal volume with both neuropsychological score and chronological age in the chemotherapy-treated survivors. These results suggest that the hippocampal changes might potentially be indicative of a neural basis for cognitive impairment in older long-term cancer survivors.

## Data availability statement

The datasets presented in this article are not readily available because it required institutional permission. Requests to access the datasets should be directed to BC, bechen@coh.org.

## Ethics statement

The studies involving humans were approved by the City of Hope National Medical Center IRB# 14283, PI: BC. The studies were conducted in accordance with the local legislation and institutional requirements. The participants provided their written informed consent to participate in this study.

## Author contributions

ED: Formal analysis, Software, Validation, Visualization, Writing – original draft, Writing – review & editing. FD: Formal analysis, Writing – review & editing. SP: Formal analysis, Methodology, Writing – review & editing. MS: Formal analysis, Writing – review & editing. JY: Formal analysis, Writing – review & editing. HK: Formal analysis, Software, Writing – review & editing. MR: Formal analysis, Software, Validation, Writing – review & editing. C-LS: Formal analysis, Software, Validation, Writing – review & editing. JR: Validation, Writing – review & editing. TA: Conceptualization, Data curation, Formal analysis, Funding acquisition, Investigation, Methodology, Resources, Supervision, Visualization, Writing – review & editing. WD: Conceptualization, Data curation, Formal analysis, Funding acquisition, Methodology, Resources, Supervision, Visualization, Writing – review & editing. BC: Conceptualization, Data curation, Formal analysis, Funding acquisition, Methodology, Resources, Supervision, Visualization, Writing – review & editing.

## Footnotes

1
http://fsl.fmrib.ox.ac.uk/fsl/fslwiki/FIRST
